# Diabetic Ketoacidosis and COVID-19: A Retrospective Observational Study

**DOI:** 10.7759/cureus.30895

**Published:** 2022-10-31

**Authors:** Govind Nagdev, Gajanan Chavan, Charuta Gadkari, Gaurav Sahu

**Affiliations:** 1 Department of Emergency Medicine, Jawaharlal Nehru Medical College, Datta Meghe Institute of Medical Sciences (Deemed to be University), Wardha, IND; 2 Department of Medicine, Jawaharlal Nehru Medical College, Datta Meghe Institute of Medical Sciences (Deemed to be University), Wardha, IND

**Keywords:** diabetic ketoacidosis (dka), covid and dka, hyperosmolar hyperglycaemic state, covid-19, diabetic ketoacidosis

## Abstract

Background

Diabetic ketoacidosis (DKA) is a life-threatening complication of diabetes mellitus (DM), mainly type 1 DM. DM is one of the comorbidities recognized as the predictor of the severity in COVID-19-positive patients. The severe acute respiratory syndrome coronavirus 2 (SARS-CoV-2) illness (COVID-19) has a bidirectional association with DM. DM is a state of chronic inflammatory condition and hyperglycemia that predisposes one to acquire an exaggerated form of COVID-19 infection. Moreover, in recent literature, it has been established that SARS-CoV-2 is capable of directly damaging beta cells of the pancreas, thereby inducing new-onset diabetes in previously non-diabetics. Hence, new-onset diabetes and severe metabolic consequences of pre-existing diabetes, such as DKA, are seen in COVID-19-positive patients. DKA in patients with COVID-19 may increase the risk of mortality and lead to poor prognosis.

Methods

This retrospective observational study includes 25 patients who presented to our hospital with DKA secondary to COVID-19 infection as a triggering factor. Demographic parameters, medical history, physical examination, laboratory tests including reverse transcriptase polymerase chain reaction test (RT-PCR), chest X-ray, treatment administered, clinical course, and outcomes were recorded. All data were computed and analyzed using SPSS Version 22.0 (IBM Corp., Armonk, NY, USA).

Results

Of the 25 cases, 14 were females, and a statistically significant difference was seen in the values of blood sugar (367 mg/dL), D-dimer, ferritin, blood urea nitrogen (BUN), and anion gap among males and females (p < 0.050). The males had higher mean values of blood sugar (367 mg/dL), BUN (60.63 mg/dL), D-dimer (1.09 mcg/mL), and ferritin (821.23 mcg/mL) than females, whereas females had a higher anion gap (20.85) than males. A negative correlation was seen between D-dimer and the following biochemical investigations in COVID-19 patients: serum bicarbonate, anion gap, chloride, BUN, creatinine, sodium, potassium, magnesium, and phosphorous. COVID-19 can present with atypical symptoms in patients with high blood sugar levels. Diabetics are more likely to experience effects on multiple organs compared to non-diabetic patients who mainly have lung involvement. Hence, a high degree of suspicion is essential to diagnose DKA early in COVID-19 patients.

Conclusion

These cases show that DKA can be precipitated by COVID-19 in a significant number of patients and that the presence of diabetes can also exaggerate the underlying COVID-19 infection, a bidirectional relationship. All cases were COVID-19-positive presenting with features characteristic of DKA. SARS-CoV-2 was precipitating factor of DKA. Patients with newly diagnosed diabetes or pre-existing diabetes were equally susceptible to DKA. Due to the high prevalence of both illnesses (DM and COVID-19) in our country, a high level of suspicion is required to detect DKA early and improve the outcome of COVID-19-related life-threatening hyperglycemic consequences.

## Introduction

Wuhan, Hubei province, in China, saw an outbreak of unexplained severe viral pneumonia in December 2019 [1,2]. Severe acute respiratory syndrome coronavirus 2 (SARS-CoV-2) was found in these patients suffering from pneumonia [3]. Diabetes patients are at a higher risk of experiencing serious outcomes, including mortality. Recent research has found that old age, as well as patients with medical comorbidities such as diabetes mellitus (DM), cardiovascular diseases, obesity, and hypertension, are more susceptible to severe illness and mortality in COVID-19 patients [4]. Diabetic ketoacidosis (DKA), diabetic ketosis, and hyperosmolar hyperglycemic state (HHS) are significant acute metabolic consequences of diabetes that are frequently triggered by infection. In Chinese retrospective research, 42 (6.4%) of COVID-19 patients developed ketosis, with 15 (35.7%) having diabetes. Among 15 diabetic patients, 3 (20%) had DKA [4]. COVID-19 has also been linked to acute hyperglycemic crises (DKA/HHS) in individuals with poorly controlled diabetes and newly diagnosed diabetes, according to a few case reports [5,6]. We hereby describe patients who were COVID-19-positive and presented to the Emergency Department of Acharya Vinoba Bhave Rural Hospital (AVBRH), Sawangi, Wardha, India, with DKA. In addition, we reviewed nearly every published instance of COVID-19-related DKA.

## Materials and methods

This retrospective observational study was carried out in the Emergency Department of AVBRH. Located at the center of India near Nagpur, this tertiary care rural hospital caters to the adjacent 11 districts with a cumulative population of more than 2 million. Between April 2021 and June 2021, a total of 532 patients presented to the hospital with SARS-CoV-2 infection, with reverse transcriptase polymerase chain reaction (RT-PCR) positive for COVID-19. Of these, 200 patients were identified as diabetics and those patients who were more than 17 years of age and lab-confirmed DKA status were enrolled in this study; informed consent was obtained for the same. Those who had starvation ketoacidosis or did not wish to participate in the study were excluded.

Criteria to diagnose patients as DKA were defined as plasma glucose > 250 mg/dL, arterial pH < 7.3, and/or serum HCO_3-_ < 1 8 mmol/L, and urine or serum for ketone bodies tested positive.

Study procedure

Demographics and Laboratory Measurements

Fulfilling the including and exclusion criteria, this study discusses 25 cases that were presented to the hospital in the defined time period during the COVID-19 pandemic. Demographic parameters including age, gender, and sociodemographic details, along with medical history, physical examination, laboratory tests including RT-PCR reports, chest X-ray, treatment administered, clinical course, and outcomes were recorded and updated in the online information system of the hospital. Each patient has their own unique identification number, and this number can be used to retrieve vital information and monitor the progress of the patient as well as referred for future reference.

COVID-19 Detection

RT-PCR for SARS-CoV-2 was used for the detection of COVID-19 in nasal and throat swabs. The Central Clinical Laboratory of AVBRH was accountable for the detection of COVID-19 in the swabs of the study patients. The laboratory has been authorized by the Indian Council of Medical Research (ICMR) to conduct the testing for COVID-19. RT-PCR was done by QuantStudio 5 using the COVID-19 one-step RT-PCR kit. The kit consisted of COVID-19 enzyme mix (lyophilized), COVID-19 primer probe mix, an enzyme mix buffer, COVID-19 PCR positive control, and COVID-19 negative control (diethylpyrocarbonate (DEPC) treated H_2_O).

Data Analysis

Patient details and lab reports were collected and tabulated in a Microsoft Excel file format. All data were computed and analyzed using SPSS Version 22.0 (IBM Corp., Armonk, NY, USA). Depending on whether the data were parametric or non-parametric, appropriate statistical tests of significance were applied. A p-value of less than 0.05 was considered statistically significant for all the tests used.

## Results

These cases show COVID-19 can be a precipitating factor for DKA in a significant number of patients. Patients with newly diagnosed diabetes or pre-existing diabetes were equally susceptible to DKA. Due to the high prevalence of COVID-19 and diabetes, a high level of suspicion is required to detect DKA in a timely manner in order to improve the outcome of COVID-19-related DKA.

On comparison of the investigations done in COVID-19 patients with respect to gender, a statistically significant difference was observed in the values of blood sugar (367), D-dimer, ferritin, blood urea nitrogen (BUN), and anion gap among males and females. The males had higher mean values of blood sugar (367 mg/dL), BUN (60.63 mg/dL), D-dimer (1.09 mcg/mL), and ferritin (821.23 mcg/mL) than females, whereas females had a higher anion gap (20.85) than males (Table 1).

**Table 1:** Comparison of gender distribution of the investigations among COVID-19 patients *Significant difference observed (p < 0.05) BUN, blood urea nitrogen

Investigations	Gender	N	Mean	Std. deviation	Sig. (two-tailed) p-value
pH	Male	11	7.17	0.13	0.931
	Female	14	7.17	0.12	
Serum bicarbonate	Male	11	12.90	2.80	0.837
	Female	14	12.67	2.85	
Blood sugar	Male	11	367.63	106.00	0.047*
	Female	14	355.85	59.22	
Anion gap	Male	11	18.36	2.61	0.048*
	Female	14	20.85	4.45	
Chloride	Male	11	102.8	4.35	0.095
	Female	14	103.5	5.43	
Urine ketone	Male	11	2.55	0.82	0.669
	Female	14	2.71	1.06	
BUN	Male	11	60.63	70.93	0.032*
	Female	14	39.57	31.80	
Creatinine	Male	11	2.03	2.98	0.275
	Female	14	1.11	0.73	
HbA1c	Male	11	8.16	1.12	0.339
	Female	14	8.48	2.08	
Sodium	Male	11	134.09	5.39	0.223
	Female	14	137.21	6.73	
Potassium	Male	11	4.40	0.63	0.211
	Female	14	4.46	0.74	
Calcium	Male	11	8.15	0.83	0.586
	Female	14	8.35	0.89	
Magnesium	Male	11	2.19	0.22	0.877
	Female	14	2.21	0.45	
Phosphorous	Male	11	4.28	0.64	0.251
	Female	14	4.01	0.49	
D-dimer	Male	11	1.09	.046	0.035*
	Female	14	0.88	0.15	
Ferritin	Male	11	821.23	293.65	0.021*
	Female	14	620.00	265.82	

Table 2 shows mean values of all laboratory investigations observed in COVID-19 patients. Mean pH value was 7.17, which indicates metabolic acidosis. Mean bicarbonate value was 12.77 mEq/L, which is less than 18 mEq/L and fits under criteria of DKA. Also, 361.04 mg/dL and 19.760 mEq/L were mean value of random blood sugar (RBS) and anion gap respectively, fulfilling criteria of DKA. Mean value of serum ferritin in all patients was high, i.e., 708.54.

**Table 2:** Mean values of the investigations observed among COVID-19 patients BUN, blood urea nitrogen; BMI, body mass index

Investigations	N	Minimum	Maximum	Mean	Std. deviation
pH	25	6.90	7.31	7.17	0.12
Serum bicarbonate	25	8.00	17.00	12.77	2.77
Blood sugar	25	270.00	625.00	361.04	81.34
Anion gap	25	13.00	31.00	19.760	3.89
Chloride	25	92.00	110.00	103.24	4.90
Urine ketone	25	1.20	5.00	2.64	0.95
BUN	25	13.00	257.00	48.84	52.52
Creatinine	25	0.40	10.80	1.52	2.05
HbA1c	25	6.53	14.00	8.34	1.70
Sodium	25	121.00	149.00	135.84	6.26
Potassium	25	3.20	5.70	4.44	0.68
Calcium	25	6.20	9.90	8.26	0.85
Magnesium	25	1.50	3.00	2.20	0.36
Phosphorous	25	3.20	5.60	4.13	0.56
D-dimer	25	0.60	2.30	0.97	0.34
Ferritin	25	25.00	1058.00	708.54	290.85
BMI	25	18.00	27.00	22.67	3.14

Table 3 shows variability in laboratory values with respect to medical history of patients. A significant difference was observed in serum bicarbonate, RBS, anion gap, urine ketone, BUN, creatinine, potassium, calcium, and phosphorus since p-value is <0.05.

**Table 3:** Investigation in association with a medical history of COVID-19 patients *Significant difference observed (p<0.05) BUN, blood urea nitrogen; CKD, chronic kidney diseases; DM, diabetes mellitus

Investigations	Nil	Type 1 DM	Type 2 DM	Hypertension	Hypertension with type 2 DM	CKD and DM	Chi-square p-value
Serum bicarbonate	10.7	11.25	13.14	13.92	12.00	16.00	0.022*
Blood sugar	327.25	366.00	352.2857	355.25	625.00	320.00	0.025*
Anion gap	19.00	17.75	20.7	20.5	16.00	22.00	0.051*
Chloride	104.25	106.25	102.57	102.12	106.00	98.00	0.239
Urine ketone	2.50	2.25	2.57	3.00	3.00	2.00	0.042*
BUN	23.75	32.5	25.57	60.25	78.00	84.00	0.001*
Creatinine	0.72	0.82	0.91	1.625	1.6	10.8	0.001*
HbA1c	8.57	8.7	8.45	8.01	9.00	6.80	0.393
Sodium	136.5	135.25	136.42	135.00	134.00	136.00	0.831
Potassium	3.8	4.8	4.45	4.6	4.2	4.00	0.042*
Calcium	8.3	8.7	8.62	8.02	7.5	6.2	0.022*
Magnesium	1.87	2.3	2.2	2.2	2.5	2.2	0.236
Phosphorous	3.7	4.1	3.9	4.2	5.00	5.6	0.003*
D-dimer	0.92	1.00	1.15	0.86	0.80	0.95	0.226
Ferritin	588.7	585.4	805.1	674.8	974.2	932.4	0.170

COVID-19 can present with atypical symptoms in patients with high blood sugar levels. People with diabetes are more likely to experience effects on multiple organs compared to non-diabetic patients who mainly have lung involvement. Hence, in individuals with DM, it is prudent to consider a low threshold for COVID-19 infection screening. On observation, there was a negative correlation of D-dimer with serum bicarbonate, Ph, anion gap, BUN, creatinine, and potassium. Angiotensin-converting enzyme 2 (ACE2) is a key enzyme in the renin-angiotensin-aldosterone system (RAAS). It is accountable for converting angiotensin II into angiotensin. ACE2 is highly expressed in the lungs and pancreas. It is through this route that SARS-CoV-2 enters the body. ACE2 expression is reduced after the virus complex is endocytosed. There are two conceivable outcomes from these interactions. First, SARS-CoV-2 infection of islet cells of the pancreas may aggravate beta cell destruction. Secondly, after the viral invasion, it impedes insulin secretion due to the downregulation of ACE2, leading to unopposed angiotensin II. These two mechanisms cause abrupt loss of beta cell function of the pancreas, attributing to DKA.

On determining the association of D-dimer with the biochemical investigations in COVID-19 patients, a negative correlation was seen with the serum bicarbonate, anion gap, chloride, BUN, creatinine, sodium, potassium, magnesium, and phosphorous (Table 4).

**Table 4:** Investigation of association between blood sugar and D-dimer of COVID-19 patients BUN, blood urea nitrogen

	Blood sugar		D- dimer	
Investigations	Pearson Correlation	Sig	Pearson Correlation	Sig.
Serum bicarbonate	-0.56	0.790	-0.34	0.093
pH	-0.12	0.540	0.21	0.301
Anion gap	-0.22	0.283	-0.34	0.093
Chloride	0.30	0.135	-0.21	0.296
Urine ketone	0.46	0.827	0.085	0.685
BUN	-0.16	0.419	-0.05	0.787
Creatinine	-0.16	0.440	-0.48	0.820
HbA1c	0.31	0.129	0.16	0.443
Sodium	136.5	0.801	-0.37	0.069
Potassium	-0.54	0.799	-.03	0.885
Calcium	0.026	0.903	0.15	0.447
Magnesium	0.15	0.468	-0.11	0.602
Phosphorous	0.22	0.275	-0.12	0.549
Ferritin	0.33	0.103	0.18	0.380

## Discussion

In this study, we describe 25 patients who had DKA secondary to COVID-19, including diagnosed and undiagnosed patients with diabetes. The effect of DM on COVID-19 severity and severe metabolic complications of pre-existing diabetes, such as DKA and HHS in COVID-19 patients along with the prevalence of new-onset diabetes in COVID patients, presents difficulty in clinical management [6]. DKA is an acute life-threatening complication of DM that occurs predominantly in insulin-dependent DM. DKA is caused by a lack of insulin and a rise in counterregulatory hormones, both of which favor the formation of ketones. Interleukin-6 (IL-6) levels have been reported to be greater in both COVID-19 and DKA, suggesting that this may be a key prognostic factor [7]. Diagnosis of DKA constitutes RBA > 250 mg/dL, anion gap > 10-12 mEq/L, pH < 7.3 with moderate ketonuria or ketonemia, and bicarbonate level < 18 mEq/L.

Severe DKA episodes leading to hospitalization in an emergency department in our cases may have been caused by COVID-19's triggering effect on diabetes. The exact pathogenesis behind this is yet to be determined. Inflammatory cytokines produced during viral infection, on the other hand, have been associated with the disease.

Interactions between RAAS and SARS- CoV-2 may give another pathophysiological explanation for DKA [8]. ACE2 is a key enzyme in RAAS. It is accountable for converting angiotensin II into angiotensin. ACE2 is highly expressed in the lungs and pancreas. It is through this route that SARS-CoV-2 enters the body. ACE2 expression is reduced after the virus complex is endocytosed [8,9]. There are two conceivable outcomes from these interactions. First, SARS-CoV-2 infection of islet cells of the pancreas may aggravate beta cell destruction [10]. Secondly, after the viral invasion, it impedes insulin secretion due to the downregulation of ACE2 leading to unopposed angiotensin II [11]. These two conditions may have had a role in our patients' abrupt loss of pancreatic beta cell function and the onset of DKA. Management of DKA can be complicated because of the relationship between SARS-CoV-2 and RAAS. Acute respiratory distress and cerebral edema can be adverse outcomes of excessive fluid resuscitation. As a result, in these patients, appropriate hydration control is important. Furthermore, because angiotensin II stimulates aldosterone secretion, the risk of hypokalemia is raised, necessitating higher supplementation of potassium so that intravenous insulin flows continuously to prevent ketone generation. Few reports of DKA in COVID-19 have been published to our knowledge.

According to new evidence, diabetics are at a higher risk of complications, including mortality, among COVID-19 patients [12]. According to a clinical analysis from China including 1,099 validated COVID-19 patients, diabetes was the second most common comorbidity (16.2%) among severe 173 cases [13,14]. Although there is not enough evidence to link diabetes to bad outcomes in COVID-19 patients, metabolic acidemia leads to decreased cardiac contractility, affects oxygenation, and results in vital organ dysfunction [15,16].

## Conclusions

In patients with pre-existing or newly diagnosed diabetes, COVID-19 may impair pancreatic beta cell activity and trigger DKA. As DM is highly prevalent in our country and COVID-19 superimposed, to enhance the outcome of COVID-19-related acute hyperglycemic consequences, a high degree of suspicion is essential to diagnose DKA early. We conclude that aggressive management starting from rapid primary survey, investigations, rapid fluid therapy, and electrolyte balance, with close monitoring in an ED setting and timely further intervention with the available treatment may lead to improved prognosis.
